# A Possible Common Neurophysiologic Basis for MDD, Bipolar Disorder, and Schizophrenia: Lessons from Electrophysiology

**DOI:** 10.3389/fpsyt.2016.00094

**Published:** 2016-06-01

**Authors:** Goded Shahaf

**Affiliations:** ^1^BrainMARC LTD, Yoqneam, Israel

**Keywords:** drive reduction, EEG, ERP, MDD, bipolar disorder, schizophrenia

## Abstract

There is ample electrophysiological evidence of attention dysfunction in the EEG/ERP signal of major depressive disorder (MDD), bipolar disorder, and schizophrenia. The reduced attention-related ERP waves show much similarity between MDD, bipolar disorder, and schizophrenia, raising the question whether there are similarities in the neurophysiologic process that underlies attention dysfunction in these pathologies. The present work suggests that there is such a unified underlying neurophysiologic process, which results in reduced attention in the three pathologies. Naturally, as these pathologies involve different clinical manifestations, we expect differences in their underlying neurophysiology. These differences and their subtle manifestation in the ERP marker for attention are also discussed. MDD, bipolar disorder, and schizophrenia are just three of multiple neuropsychiatric disorders, which involve changes in the EEG/ERP manifestations of attention. Further work should expand the basic model presented here to offer comprehensive modeling of these multiple disorders and to emphasize similarities and dissimilarities of the underlying neurophysiologic processes.

## Deviation in the Electrophysiological Marker of Attention

Various psychopathologies, such as major depressive disorder (MDD), bipolar disorder, and schizophrenia, involve significant dysfunction of attention-related processes (1–3). There is ample electrophysiological evidence for this attention dysfunction in the EEG/ERP signal of MDD, bipolar disorder, and schizophrenia (4–6). The reported evidence involves attention-related ERP waves of longer latencies (e.g., P300) (7). Some deviations are reported also for earlier ERP waves, but as we showed in previous work, these may also be attributed to attention dysfunction (8, 9).

There is a basic similarity of reduced attention-related ERP waves between MDD, bipolar disorder, and schizophrenia. This raises the question whether there is also a similarity with regard to the neurophysiologic process that underlies attention dysfunction in these pathologies. The present work suggests that there is such a unified underlying neurophysiologic process that causes reduced attention in the three pathologies. Naturally, as these pathologies involve different clinical manifestations, we expect differences in their underlying neurophysiology. These differences, and their subtle manifestation in the ERP marker for attention, are also discussed.

## A Basic Neurophysiologic Model for MDD

Ample electrophysiological evidence has been accumulating regarding reduced attention in MDD. Reduced P3 amplitude, delayed latency, or both are often reported (4, 10). Multiple regions and pathways, both cortical and sub-cortical, are involved in evoking P3 and attention (9). The question is: the dysfunction of which of these regions and pathways underlies the functional and electrophysiological changes in MDD?

Functional brain imaging in MDD often reveals reduced activity in the dorsolateral prefrontal cortex (DLPFC) (11). This region plays a key role in executive function, and its hypo-functionality can explain reduced attention and related ERP manifestations, as well as various hypo-functionality symptoms in MDD. But reduced activity in the DLPFC is not specific to MDD and, therefore, the question regarding its underlying cause remains.

Another finding that emerges from functional brain imaging in MDD involves the other division of the prefrontal cortex, the ventromedial prefrontal cortex (VMPFC). It appears that in MDD the activity of the VMPFC increases in contrast to the decreased activity of the DLPFC (12). The VMPFC is known to exert an inhibitory effect on the output of the basolateral amygdala nuclei (13, 14). This amygdalar complex plays a key role in the attentive response to stimuli (9), and may be viewed as a gatekeeper between the more posterior perceptual regions and the attentive response involving prefrontal regions. The amygdalar complex seems to direct the activation of the prefrontal regions to relevant stimuli that are perceived as significant. Therefore, the top-down inhibition of this complex by the VMPFC can be expected to reduce attention and its electrophysiological manifestations. In the normal interaction between the prefrontal cortices and the amygdala, there appears to be an inherent preference of VMPFC over DLPFC activation (15), which promotes response inhibition, but still enables a response in the DLPFC to relevant stimuli. In MDD, repetitive stressing stimuli might increase the preference for VMPFC activation owing to plasticity changes in the connectivity between the amygdalar complex and the VMPFC. There is some evidence in support of such plasticity changes under stress (16).

The above theory concerning the top-down inhibition of the output of the basolateral complex of the amygdala as the mechanism of MDD appears to contradict the reports of increased amygdalar activity in this disorder in fMRI studies (17). But fMRI findings are generally reported at the resolution of the entire amygdala, and it has been shown that different amygdalar complexes are activated in widely different patterns (18). Top-down inhibition from the VMPFC seems to induce activation in the amygdala, which can manifest as increased fMRI activity. But this activation is of inhibitory complexes, which then inhibit the output activation from the basolateral complex (19).

**Figure 1:**
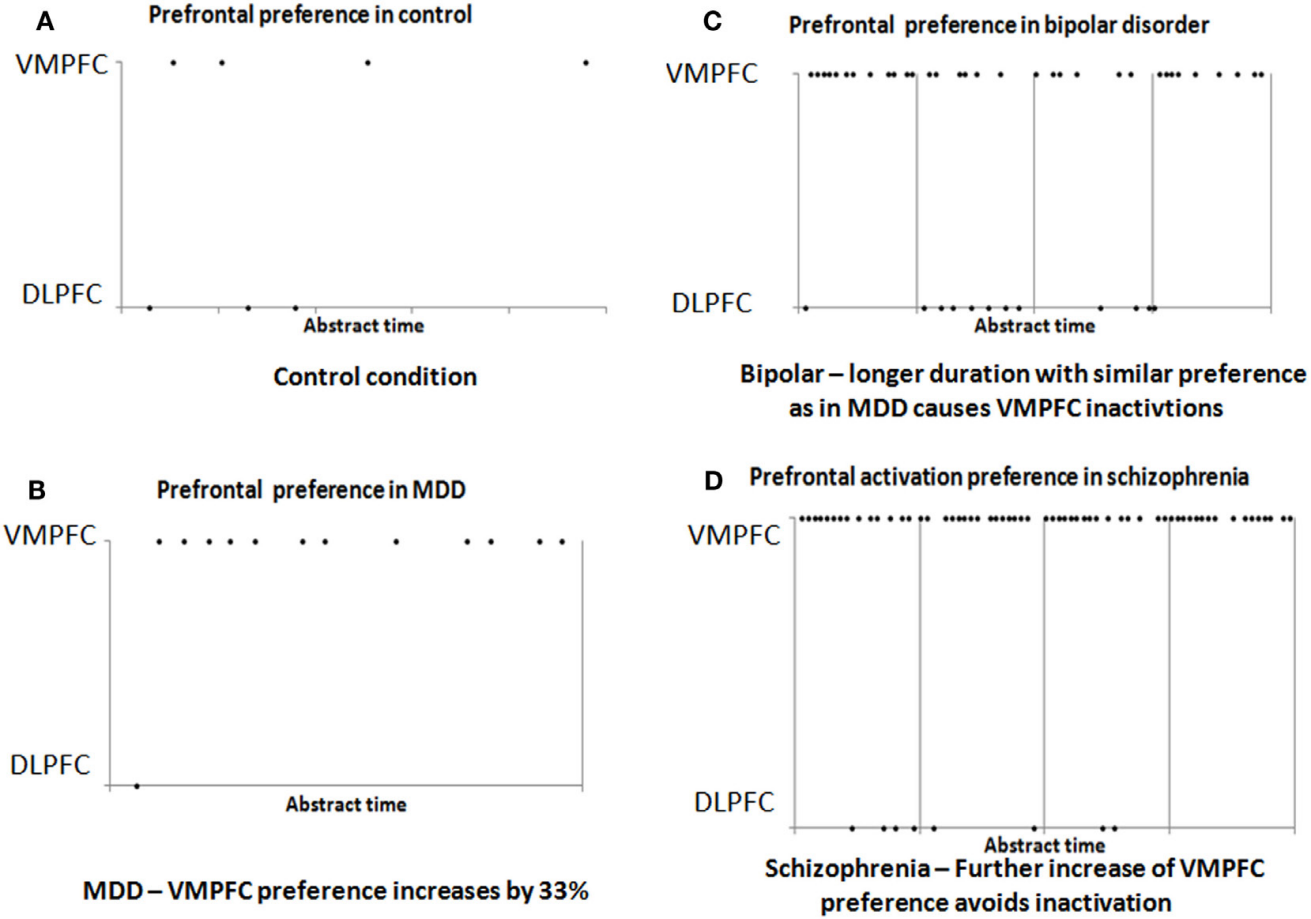
**Results of simulation of the prefrontal cortex activation in the control (A) and MDD (B) conditions**. Note the greater activation of VMPFC, which results from an increase of ~33% in the strength of input from the amygdala, without any other change. The increased activation results in reduced DLPFC activation. **(C)** If the increased activation of the VMPFC continues for a sufficiently long duration, it enters periods of inactivation. In these periods, lateral prefrontal cortex activation is increased. **(D)** Further increase of ~33% (beyond that in Figures 1 and 2) in the association strength of the input to the VMPFC from the amygdala reduces its periodic inactivation (and the periodic activation of the lateral prefrontal cortex).

We previously reported the development of a simulation tool that enables the neurophysiologic modeling of behavioral functions. The simulator contains a modular specification of brain regions, each region containing modular neuronal networks, which are its elementary units of representation. The simulator enables parametric selection of most infrastructure and anatomical constraints to support the evaluation of various theories regarding connectivity and flow among regions, and their effect on function (9). We suggested a detailed model for the process that was described in general terms above, from stimulus sensation, through gate-keeping at the amygdala, to the path to motor response. Below we use this simulation to demonstrate the elementary feasibility of our model for the three psychopathologies. Figures 1A,B shows the degree of activation of the two prefrontal regions in the control condition and after increase of ~33% of the strength by which the amygdalar complex activates the VMPFC (which represents the plasticity suggested above for depression). As shown in Figures 1A,B, such an increment reduces significantly the response in the DLPFC because of reciprocal inhibition of the amygdalar complex. This is consistent with the electrophysiological findings described above for MDD.

## Extension of the Model to Bipolar Disorder

Reduced ERP attention markers are also reported consistently for bipolar disorder. Several studies have reported that attention reduction is greater in bipolar disorder than in MDD (20), and it is greater during manic episodes than during depressive ones (5) (bipolar disorder contains alternating periods of depression and mania). Earlier, we suggested that depression may result from over-activation of the VMPFC by the amygdalar complex. When a neuronal network is activated at sufficient intensity for a sufficient duration, it tends to generate periods of inactivation (21, 22). The basic preference of VMPFC over DLPFC (15) seems to be enhanced in depression. In return, the VMPFC inhibits the amygdalar complex and reduces activation of the lateral prefrontal cortex. But if the VMPFC response enters a period of relative inactivation, its inhibition of the amygdalar complex is reduced and activation of the lateral prefrontal cortex is more likely.

Figure 1C illustrates this dynamic and shows the effect of continuous activation of the simulation model, while the strength by which the amygdalar complex activates the VMPFC is increased by 33% from the control level. The figure shows that if the stimulation of the model is maintained for a sufficient duration, it evokes periodic shifts in which the lateral prefrontal cortex is at times inhibited and at times activated. This can explain the clinical dynamics in bipolar disorder. Note that during the bipolar cycle, even during the manic phase, the activation of the VMPFC is strong and, therefore, the global inhibition of the DLPFC is also strong. Nevertheless, there are still enough periods of greater activation of the lateral cortex, which could underlie the manic behavior. The overall stronger global inhibition of the DLPFC can explain the reduced electrophysiological activity reported during the manic phase.

In keeping with the overall global inhibition, fMRI and PET findings do not show consistently greater activation of the DLPFC during manic episodes (23); rather, they show greater activation of other lateral prefrontal regions, which are further downstream in the executive-motor hierarchy in the manic episodes than in the depressive ones (24). It is possible that VMPFC is still sufficiently active to inhibit the more demanding lasting recruitment of upstream DLPFC regions, but downstream shorter motor-oriented regions may periodically escape inhibition. This differentiation may manifest in more impulsive behavior, which seems to accord with the clinical manifestation.

## Further Extension of the Model to Schizophrenia

Schizophrenia also involves reduced ERP attention markers, and it appears that the markers are more reduced in schizophrenia than both in MDD and in bipolar disorder (6, 25). The reduction seems to be caused by further reduction in DLPFC activity (26, 27).

Psychotic episodes in schizophrenia are often preceded by periods of negative symptoms that resemble depression. We suggested above that such depressive symptoms may be caused by over-activation of the VMPFC by the amygdalar complex, which reduces activation of the DLPFC. We further suggested that sufficient intensity for a sufficient duration of such VMPFC over-activation can produce periods of inactivation, which manifest as relatively increased activation of the lateral prefrontal cortex. It was suggested that this pattern underlies bipolar disorder. But it is possible to stimulate a given neuronal network at an intensity that avoids such inactivation periods (22). In the case of the VMPFC, such strong stimulation may prevent periods of increased lateral prefrontal activation, and instead lead to greater amygdalar suppression. Figure 1D illustrates this effect, showing that further increase of the amygdalar effect upon the VMPFC (altogether a 66% increase over the control condition) reduces the inactivation periods and thereby maintains inhibition of the DLPFC.

Another electrophysiological finding, which distinguishes schizophrenia from MDD and bipolar disorder, is the significant reduction of attention markers over more posterior perception-related regions (28, 29). These posterior regions also seem to receive significant reciprocal excitatory input from the amygdalar complexes, which are inhibited by the VMPFC (30–32). It has been suggested that in schizophrenia this amygdalar-perception excitation is reduced (33). But if the amygdalar complex is strongly inhibited, we can expect reduced top-down activation of the perception regions in response to standard stimuli. Such reduced activation may in turn result in increased responsiveness to less relevant activations because of increased sensitivity caused by reduced activity (34). Such less relevant activations can be evoked by top-down connections from the VMPFC to the perception regions (35).

**Figure 2:**
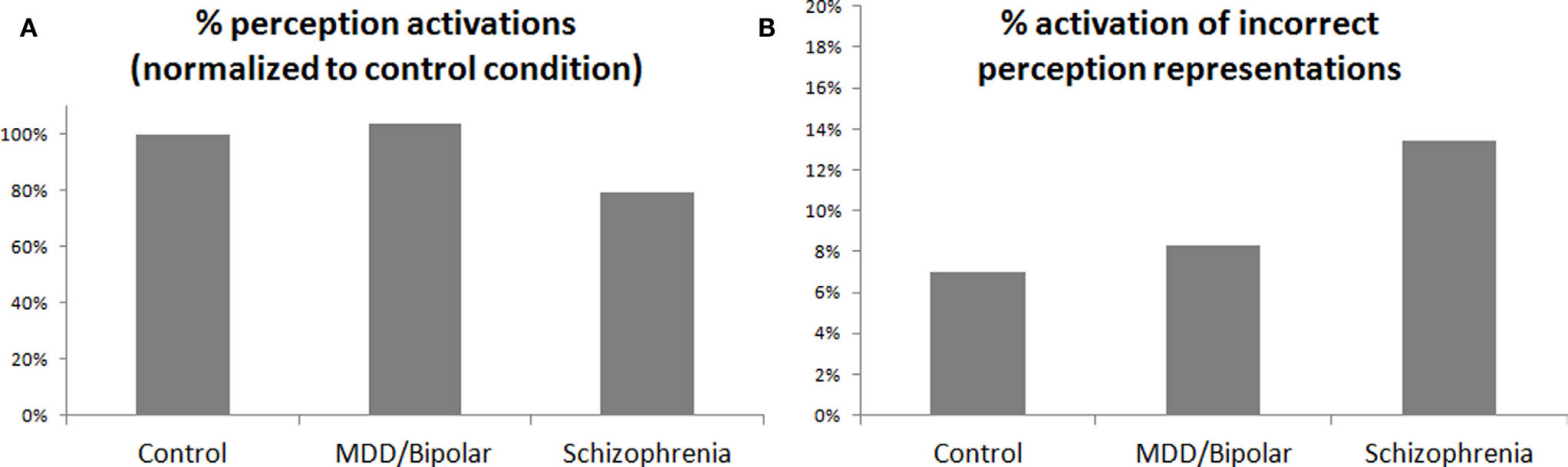
**(A)** Percentage of activation of perception representations by stimuli normalized to the control condition. **(B)** Percentage in which a given stimulus evoked representations of other (wrong) stimuli in the simulated secondary perception region. Note that the only difference between the three simulated conditions is in the excitatory effect of the amygdala upon the VMPFC. This effect is 33% larger in MDD/bipolar disorder than in the control condition, and 66% larger in schizophrenia than in the control condition. The presented effect on the perception region is the result of reciprocal inhibition of the VMPFC on the relevant amygdalar complex, which in turn reduces excitation of the perception region.

**Figure 3:**
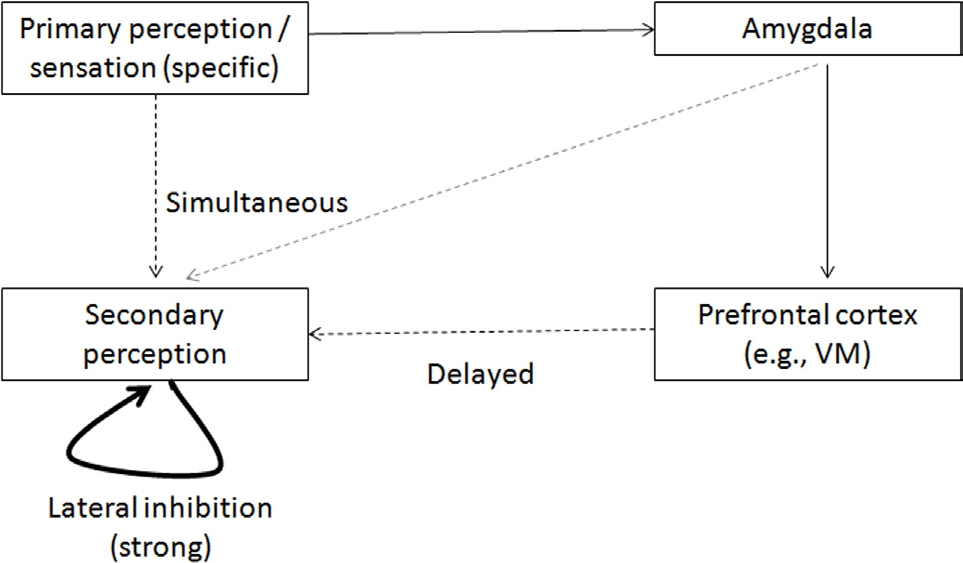
**Model of activation of secondary perception representation**. A specific stimulus evokes near-threshold (broken line) activation of its related representation in the secondary perception region. It also activates simultaneously the amygdala, evoking a top-down activation of the secondary perception representation, which is weaker and not specific (gray broken line). The two activations overlap sufficiently in time to enable threshold crossing of the specific activation in the secondary perception region. This activation inhibits the possibility of competitive activations in this region (through lateral inhibition). But if the top-down amygdalar activation is disabled (owing to its inhibition in the schizophrenia condition), the specific representation in the secondary perception region is activated less and, therefore, there is less lateral inhibition and greater likelihood of erroneous activation, which might originate, for example, from the VMPFC, and could activate other representations.

Figure 2A presents the simulated degree of activation of perception representations evoked by repetitive stimuli, normalized to the control activation level. It is possible to see the reduced activation in the schizophrenia condition. Figure 2B presents the percentage of activation of erroneous perception representations in the simulation, specifically, representations related to other stimuli and not to the stimulus that was presented to the simulation. Figure 2B shows that such erroneous activations are more prevalent in the schizophrenia condition. As noted above, only the degree of activation of the VMPFC by the amygdala was increased between the simulation conditions, but sufficient increase of this activation leads to increased reciprocal inhibition of the relevant amygdala complexes by the VMPFC. If this inhibition is sufficiently strong, as in the simulated schizophrenia condition, it leads to reduced excitation of the perception regions by the amygdala and, thus, to the results shown in Figures 2A,B. The increase in erroneous activation in the schizophrenia condition stems from the excitation scheme presented in Figure 3, which is based on the literature [(9), and the literature cited above regarding the interaction of the amygdala with the VMPFC and with perception regions]. In other conditions, there is sufficient co-activation of the perception regions both bottom-up, from primary sensory regions, and top-down, from the amygdala. But if top-down activation is reduced, as suggested for schizophrenia, it results in reduced threshold crossing and reduced activation of the correct perception representations. This, in turn, reduces the lateral inhibition within the perception regions and enables erroneous activation.

This erroneous perception activation resulting from the increased amygdalar inhibition might be the underlying mechanism of hallucinations and delusions in schizophrenia. The notion that reduced activation of sensation and perception regions may underlie hallucinations is well established (36, 37). It was further demonstrated that stimulus activation of the perception regions is reduced in schizophrenia (38). Therefore, it has been suggested that top-down deafferentation is what produces hallucinations and delusions in schizophrenia, and could be compared with bottom-up sensory deafferentation in sensory-deprivation conditions (39).

In sum, the electrophysiological findings in MDD, bipolar disorder, and schizophrenia, as well as other major clinical symptoms may be explainable by increased activation of the VMPFC by the amygdala. The increased activation may be the result of stress-induced plasticity. Note that in the case of schizophrenia, such increased activation cannot be proved or disproved by standard functional imaging modalities, such as fMRI. Indeed, fMRI studies do not report increased VMPFC activation (40, 41). But fMRI measures summarize activity over hundreds of milliseconds, which stems not only from the immediate response to the stimulus but maybe also more from reverberating working memory activation. Working memory, however, involves interaction between the prefrontal and perception regions, and as we suggested, it appears that in schizophrenia perception regions are inhibited, which can hinder working memory and reduce its manifestation in fMRI. It seems that better temporal resolution is essential, and that it can be achieved with EEG/ERP attention-related waves. The consistent finding of reduced attention-related EEG/ERP waves, which is prominent in MDD when compared with control subjects, with further reduction in bipolar disorder and again in schizophrenia, is of interest despite poor spatial localization of the underlying activity. Above we modeled this underlying activity on the basis of established evidence from neurophysiology and from functional neuroanatomy.

## Stress-Induced and Self-Perpetuating Dynamics

Stress is a key factor in clinical deterioration in MDD (42), bipolar disorder (43), and schizophrenia (44). The enhanced activation of the VMPFC, stressing and negative emotion stimuli, and the resulting inhibition of relevant amygdala activity are well documented (45–47). As suggested above, this enhanced activation may produce plasticity changes, preferring the stimulus activation of the VMPFC over the DLPFC (16). There is reason to believe that the preference of VMPFC and its top-down effect can explain the electrophysiological as well as the central clinical characteristics of MDD, bipolar disorder, and schizophrenia. As suggested above, differences in these characteristics between the disorders are the result of mere intensification of the stress-induced preference of the VMPFC, which may be larger in bipolar disorder than in MDD, and larger yet in schizophrenia.

But VMPFC preference and DLPFC inhibition seem to reduce the ability of patients to explore more adaptive solutions for their stressors. This is manifested in reduced cognitive functioning during clinical deterioration (3, 48–51). The reduced cognitive functioning is manifest in MDD, and as expected from the above model, even more so in bipolar disorder and in schizophrenia. The reduced ability to explore adaptive solutions appears to contribute to the perpetuation of the pathological solution of amygdala inhibition by increased VMPFC preference.

Based on the model suggested above, it is possible to ascribe the greater cognitive dysfunction in bipolar disorder than in MDD (48, 49) not only to greater amygdalar inhibition but also, as suggested above, to the preference of inappropriate responses caused by the preference of downstream activation of the lateral prefrontal cortex during the manic phase.

Similarly, the greater cognitive dysfunction in schizophrenia (50, 51) may be ascribed not only to even greater amygdalar inhibition but also to reduction and distortion of perception during the psychotic phase. In a sense, by further reducing adaptive exploration, both manic behavior and psychoses appear to contribute even more to the self-perpetuation of the pathology, as a dysfunctional solution to the underlying stressors. Thus, bipolar disorder and schizophrenia may require more intensive treatment intervention.

The reduced exploration after the onset of deterioration leads to maladaptive behavior, which results in self-perpetuation and snowball intensification of the clinical condition. It also seems that the psychopathologies also self-perpetuate between deterioration episodes. An episodic deterioration may offer temporary reduction of the response to stress, but it is a stressor itself and leads to stressing attitude changes toward the patients by their environment (52, 53). Continuous stress may lead to another clinical deterioration and prevent the adoption of more adaptive solutions for the stressors.

Strong or stressing stimuli drive neuronal systems to explore for effective solutions, which may remove the stressors and, thus, stabilize by reducing further exploration (54, 55). Much was learned about the neurophysiologic embodiment of this exploration (56) and stabilization of learning (57), yet the principle formation and stabilization of solutions, which relieve stress, seem robust regardless of the precise details of its implementation. It seems that when effective solutions for the stressors are not found, dysfunctional solutions may form and become stabilized by reducing further exploration. In a sense, these dysfunctional solutions remove the stressing drive internally by reducing attention to it. At times, they also remove it for a while from the external environment, as demands from the patient are reduced during the episode of clinical deterioration.

In the present work, we showed that MDD, bipolar disorder, and schizophrenia may be such dysfunctional drive-reduction methods, which share a common underlying neurophysiologic basis: the preference of VMPFC. Other psychopathologies and stress-related disorders, such as anxiety disorders (58), substance abuse (59), chronic pain disorders [e.g., migraine (60)], and so on, may also involve stress-induced changes in attention-related EEG/ERP waves. Nevertheless each such disorder may involve a somewhat different underlying neurophysiologic mechanism. It seems valuable to expand in future work the basic model presented here and to aim at a comprehensive model for these multiple disorders, emphasizing neurophysiologic similarities and dissimilarities.

At least for the three disorders discussed in this work, the ERP attention markers are sensitive to the patient’s condition. When the condition improves, the marker tends more toward the normalized amplitude and latency; the opposite occurs when the condition deteriorates (5, 61–64). The ERP markers seem to be highly sensitive to change in clinical condition, to the degree that variations allegedly predict changes in subjectively reported measures weeks in advance (65–68).

## General Implications

The purpose of this paper was to present the theory. The theory suggests that MDD, bipolar disorder, and schizophrenia are manifestations of a single neurophysiologic dysfunction, which differs in the different conditions quantitatively rather than qualitatively. The theory implies that greater flexibility should be applied in diagnosis criteria and treatment selection. The neuroanatomical localization of the dysfunction could enable effective localization of various electromagnetic treatments, such as deep brain stimulation and TMS.

Emphasis on the role of stress and the possible snowball dynamics of deterioration suggest that early intervention is important (69). The intervention may be behavioral or cognitive, empowering the patient to find more effective ways to reduce the driving stress (70).

The studies cited above suggest that EEG/ERP attention-related markers are sensitive to early clinical change, enabling early identification of response to treatment or the lack thereof (4, 71), but also early identification of clinical deterioration. It often takes several weeks before response to treatment or clinical deterioration is determined. It may be possible to derive an easy to use electrophysiological marker for early intervention based on the attention-related markers. Such a marker may also evolve to guide in real time both behavioral and focused electromagnetic treatments (72).

Related implications may be drawn with regard to multiple other neuropsychiatric disorders, which, as suggested above, may also be modeled as dysfunctional solutions for stress. But this requires meticulous neurophysiologic modeling of each such disorder.

## Author Contributions

The author confirms being the sole contributor of this work and approved it for publication.

## Conflict of Interest Statement

The author declares that the research was conducted in the absence of any commercial or financial relationships that could be construed as a potential conflict of interest.
